# Non-invasive imaging reveals convergence in root and stem vulnerability to cavitation across five tree species

**DOI:** 10.1093/jxb/eraa381

**Published:** 2020-09-18

**Authors:** Jennifer M R Peters, Alice Gauthey, Rosana Lopez, Madeline R Carins-Murphy, Timothy J Brodribb, Brendan Choat

**Affiliations:** 1 Hawkesbury Institute for the Environment, Western Sydney University, Richmond, NSW, Australia; 2 Oak Ridge National Laboratory, Climate Change Science Institute & Environmental Science Division, Oak Ridge, TN, USA; 3 Departamento de Sistemas y Recursos Naturales. Universidad Politécnica de Madrid, Ciudad Universitaria, Madrid, Spain; 4 School of Biological Sciences, University of Tasmania, Hobart, TAS, Australia; 5 University of Cambridge, UK

**Keywords:** Cavitation, drought, embolism, microCT, roots, xylem

## Abstract

Root vulnerability to cavitation is challenging to measure and under-represented in current datasets. This gap limits the precision of models used to predict plant responses to drought because roots comprise the critical interface between plant and soil. In this study, we measured vulnerability to drought-induced cavitation in woody roots and stems of five tree species (*Acacia aneura*, *Cedrus deodara*, *Eucalyptus crebra*, *Eucalytus saligna*, and *Quercus palustris*) with a wide range of xylem anatomies. X-ray microtomography was used to visualize the accumulation of xylem embolism in stems and roots of intact plants that were naturally dehydrated to varying levels of water stress. Vulnerability to cavitation, defined as the water potential causing a 50% loss of hydraulic function (P_50_), varied broadly among the species (–4.51 MPa to –11.93 MPa in stems and –3.13 MPa to –9.64 MPa in roots). The P_50_ of roots and stems was significantly related across species, with species that had more vulnerable stems also having more vulnerable roots. While there was strong convergence in root and stem vulnerability to cavitation, the P_50_ of roots was significantly higher than the P_50_ of stems in three species. However, the difference in root and stem vulnerability for these species was small; between 1% and 31% of stem P_50_. Thus, while some differences existed between organs, roots were not dramatically more vulnerable to embolism than stems, and the differences observed were less than those reported in previous studies. Further study is required to evaluate the vulnerability across root orders and to extend these conclusions to a greater number of species and xylem functional types.

## Teaser

Using synchrotron-based microCT to measure drought-induced cavitation, we found little to no segmentation between root and stem vulnerability in five woody species with a wide range of xylem anatomies.

## Introduction

Hydraulic dysfunction caused by cavitation and the subsequent formation of gas emboli in xylem conduits is a primary cause of canopy dieback and plant mortality during periods of water stress imposed by drought (Tyree and Sperry, 1989; Venturas *et al.*, 2016; Choat *et al.*, 2018; Powers *et al.*, 2020). Vulnerability to cavitation varies broadly across species (Choat *et al.*, 2012) and has been quantitatively linked to mortality thresholds in woody plants (Brodribb and Cochard, 2009; Urli *et al.*, 2013; Li *et al.*, 2016). Because vulnerability to cavitation is determined by well understood physical processes and can be quantified for a given species or population, this trait holds great promise as a threshold parameter to predict drought-induced mortality in plants (Choat *et al.*, 2018). Hydraulic traits are now being incorporated into process-based models in order to improve predictions of plant response to drought (Mackay *et al.*, 2015; McDowell *et al.*, 2016; Martin StPaul *et al*., 2017). However, vulnerability to cavitation can vary between organs (e.g. stems, roots, and leaves) within a plant (Kavanagh *et al.*, 1999; Martínez-Vilalta *et al.*, 2002; McElrone *et al.*, 2004; Skelton *et al.*, 2017*a*; Rodriguez-Dominguez *et al.*, 2018), creating additional complexities in modelling hydraulic response to drought, and requires a thorough understanding of coordination of vulnerability to cavitation in different parts of the hydraulic pathway (Sperry *et al.*, 1998).

Root hydraulic traits are understudied compared with stem and leaf traits. Roots are far less accessible than above-ground plant organs, and the architectural complexity of roots creates difficulties for sample collection and standard hydraulic measurement protocols. However, roots are a critical component of the plant hydraulics pathway because they are the interface between the plant and soil, and are responsible for delivering water and nutrients to all above-ground plant tissues. As such, hydraulic failure in the root system could result in plant mortality independent of limitations in the stem and leaf tissue. In this context, a deeper understanding of root hydraulic function is essential for our ability to model plant response to water stress and predict plant survival under drought.

To date, the majority of previously published data suggests that, within a plant, root xylem is significantly more vulnerable to embolism than stem xylem and may represents a weak link in the plant hydraulic pathway during drought (e.g. Alder *et al.*, 1996; Hacke and Sauter, 1996; Mencuccini and Comstock, 1997; Kavanagh *et al.*, 1999; Martínez-Vilalta *et al.*, 2002; McElrone *et al.*, 2004; Choat *et al.*, 2005; Johnson *et al.*, 2016; Wason *et al.*, 2018). These studies emphasize the key role roots play when considering the whole-plant hydraulic system (Johnson *et al.*, 2016), but also suggest a lack of functional coordination between root and shoot xylem. Large differences in vulnerability between the two plant organs would result in high levels of embolism formation in root xylem during mild or moderate drought, while stems remained unaffected by hydraulic dysfunction. In the absence of embolism repair mechanisms (Lamarque *et al.*, 2018; Choat *et al.*, 2019), significant cavitation in the root system would translate to longer recovery times and greater investment in new root tissues when drought was relieved. In this context, plant survival during drought would be best served by adaptive coordination in which root and stem xylem exhibited more similar vulnerabilities.

Over the last decade, methodology in plant hydraulics has evolved while responding to concerns that errors and artefacts may be introduced though destructive harvesting of plant material and sample manipulation. A principle dilemma in the field of plant hydraulics is that studying a system that contains water under tension is intrinsically problematic because observation or manipulation can easily disrupt liquid in a metastable state. Traditional techniques used to estimate vulnerability to cavitation are indirect and destructive. Several studies indicate that experimental artefacts can introduce significant errors into measurement of vulnerability to cavitation. These errors occur most frequently in species with long and wide xylem vessels and relate primarily to excision of plant material under tension (Wheeler *et al.*, 2013; Torres-Ruiz *et al.*, 2015) and open vessels in segments used for centrifugation and air injection (Choat *et al*., 2010, 2016; Cochard *et al.*, 2010; Ennajeh *et al.*, 2011; Torres-Ruiz *et al.*, 2014). These artefacts are particularly relevant to roots of angiosperm species, which often have longer and wider xylem vessels than corresponding stem xylem (Zimmermann and Potter, 1982; Martinez-Vilalta *et al.*, 2002; McElrone *et al.*, 2004). Such artefacts would systematically overestimate the vulnerability of roots and could help to explain the lack of coordination particularly among angiosperms in the previous literature.

Non-invasive and *in situ* imaging methods are now becoming widely used tools to quantify changes in xylem functional status that accompany environmental stress and, in particular, to measure vulnerability thresholds in plants (Choat *et al.*, 2010; Brodersen *et al.*, 2013; Knipfer *et al.*, 2015; Torres-Ruiz *et al.*, 2015; Brodribb *et al.*, 2016; Scoffoni and Jansen, 2016; Skelton *et al.*, 2018). They are free from methodological artefacts associated with destructive hydraulic techniques and allow direct observations on living, intact plants in real-time. A number of imaging techniques have now been applied to measure vulnerability to cavitation including MRI, X-ray microtomography (microCT), and visible light camera technologies (Choat *et al*., 2010, 2016; Cochard *et al.*, 2015; Brodribb *et al.*, 2016). MicroCT has emerged as a popular reference technique to estimate vulnerability because of the high resolution, three-dimensional data volumes that can be acquired with relatively short scan times (McElrone *et al.*, 2013; Torres-Ruiz *et al.*, 2015). This provides insights into how network-level properties of the xylem may influence water transport and embolism spread (Brodersen *et al.*, 2011; Bouda *et al.*, 2019). However, the use of such techniques is extremely limited for roots, primarily due to expense and limited access to microCT and MRI facilities. Additionally, space within the hutch (the enclosure in which samples are placed for microCT imaging) can severely limit the physical size of plant material.

Three recent studies have used imaging techniques to investigate the relationship between vulnerability across plant organs: Skelton *et al.* (2017*a*) used microCT to image hydraulic failure in the vasculature of a herbaceous species (*Solanum lycopersicum*); Losso *et al.* (2019) imaged two species of tree seedlings of 15–20 cm length to investigate hydraulic function of seedling tissues; and Rodriguez-Dominguez *et al.* (2018) used optical visualization of embolism events to compare vulnerability in different organs of olive (*Olea europaea*) saplings. Each of these three studies found little or no difference between organ vulnerability to cavitation in the species examined. Although the data reported from non-invasive imaging thus far are sparse and disparate, taken together they suggest that root xylem may not be as vulnerable as previously thought.

Additional measurements are required to determine whether there are generalizable patterns in vulnerability between roots and stems and improve our understanding of drought tolerance at the whole-plant level. Non-invasive imaging techniques are particularly well suited to addressing these questions because they allow measurements to be made simultaneously on root and stem tissues, as well as providing spatial information on the dynamics of embolism formation and spread within intact plants.

Here, we determined the xylem vulnerability to cavitation of woody roots and stems of five tree species using synchrotron-based microCT. Two long-vesselled, diffuse porous eucalyptus species were measured: one native to a mesic climate (*Eucalyptus saligna* Sm.) and the other predominantly found in semi-arid landscapes (*Eucalyptus crebra* F.Muell.). *Acacia aneura* F.Muell. ex Benth is a highly drought-resistant species that grows in arid, inland environments. A North American oak species (*Quercus palustris* Münchh.) and an Asian conifer species [*Cedrus deodara* (Roxb.) G.Don] were also included to broaden the range of xylem functional types examined. These species were selected to investigate whether root and stem vulnerability differ across species that span a wide range of drought tolerance and xylem anatomical types. In this experiment, we utilized a hutch system available at the Australian Synchrotron that allowed for imaging of large potted plants and rapid repositioning of samples between root and shoot zones. We tested the hypothesis that the vulnerability of roots is significantly higher than that of stems, particularly for woody angiosperms, and examined differences in vulnerability with respect to xylem functional type.

## Materials and methods

### Plant material

Plant material was sourced from a local Sydney nursery and grown in 10 litre pots under well-watered conditions in the tunnel house on Western Sydney University Hawkesbury campus for 4 weeks prior to measurement at the Australian Synchrotron. Individuals of *A. aneura* were grown from seed on Hawkesbury campus in 25 litre soil bags. At the time of measurement, plants were 2–3 years old and ranged in height from 1.5 m to 2.5 m; stems were 10–25 mm in diameter at the scan site. For each experiment, plant material was transported to the Australian Synchrotron (Clayton, VIC, Australia) and allowed to dehydrate naturally over a period of ~5 d.

### Sample preparation and experimental dehydration

Three to five plants of each species were scanned at multiple time points (between five and nine time points) over 4–5 d of dehydration. To avoid image artefacts potentially caused by pot walls and dense soil, and to facilitate imaging of a large central root, soil was gently washed from the roots of each plant at the beginning of each experiment. This procedure accelerated drying although it did not induce additional cavitation in the large woody roots based on the initial observations at high water potential, which showed native embolism within the range exhibited by potted plants with intact soil (Table 1). The plant was centred on the rotating stage and attached, using cable ties, to a stage-mounted 70 cm vertical rod with two cross bars for support and to prevent movement during scanning. The main stem axis was scanned between 10 cm and 30 cm above the root collar, and a large woody root, generally the central tap root if evident, was selected for scanning at a site between 5 cm and 10 cm below the root collar. Scan sites were marked using correction fluid to guide repeat scans. Stem psychrometers (ICT International, Armidale, NSW, Australia) were carefully installed before the first scans without damaging the underlying xylem at the base of each stem, between the two scan sites, to monitor stem xylem water potential. Stem water potential (Ψ _X_) was also measured at the time of each scan on a leaf that had been previously enclosed in plastic wrap and aluminium foil for at least 30 min to equilibrate with stem xylem water potential using a pressure chamber (PMS Instrument Company, Albany, OR, USA). There was good agreement between the two measurements of stem xylem water potential (Supplementary Fig. S1 at *JXB* online). After the completion of scanning for each individual, we cut stems and roots to fully embolize all xylem vessels and rescanned at the same stem and root locations. These fully embolized ‘cut’ scans were used to determine total vessel counts for each plant. Cut scans were not obtained for *E. saligna* because of limitation in beamtime; for this species, total vessel counts were made using scans where both air- and water-filled vessels were clear and easy to distinguish.

**Table 1.** Xylem vulnerability to cavitation (P_12_ and P_50_) of stems and roots of five woody species

Species	Organ	P_12_ (–MPa)	P_50_ (–MPa)	Native embolism
*Acacia aneura*	Stem	6.72 [4.80, 7.66]	11.93 [11.53, 15.05]	6.5±4.7%
	Root	7.31 [6.34, 8.06]	9.64 [9.20, 10.21]	8.2±2.8%
*Cedrus deodara*	Stem	3.35 [2.77, 4.25]	6.77 [5.73, 7.43]	4.1±2.1%
	Root	3.51 [2.68, 4.37]	6.33 [5.90, 7.81]	5.1±5.1%
*Eucalyptus saligna*	Stem	3.12 [2.64, 3.57]	4.86 [4.53, 5.09]	5.3±0.2%
	Root	2.75 [2.45, 3.23]	4.82 [4.54, 5.52]	8.1±2.6%
*Eucalyptus crebra*	Stem	3.54 [3.00, 4.37]	5.63 [5.24, 6.08]	2.1±0.5%
	Root	1.63 [–, 3.55]	3.98 [2.14, 5.03]	11.3±8.1%
*Quercus palustris*	Stem	2.85 [2.19, 3.59]	4.56 [4.18, 4.91]	3.6±0.3%
	Root	1.19 [0.23, 2.01]	3.13 [1.83, 3.59]	21.1±2.5%

Estimates of P_12_ and P_50_ were generated from Weibull curve fit with 95% confidence intervals shown in brackets. Means of P_12_ and P_50_ are significantly different (*P*=0.05) between roots and stems of a species where confidence intervals do not overlap. Mean native embolism and standard error are taken from initial scans of each replicate plant scanned in a well-watered state.

### X-ray microCT

MicroCT scans were performed at the Australian Synchrotron light source in Clayton, VIC, Australia. The imaging and medical beamline (IMBL) was used with the Ruby 2 detector at an X-ray energy of 30 keV. These parameters provided a field of view of 28 mm in *X* and *Y* orientations and 20 mm in *Z* (vertical) orientation, with a reconstruction resolution of 9.9 μm per pixel. Plants were placed on a rotating stage and positioned in the beamline using a robotic arm (Kuka, KR 1000 Titan), and scanned in two locations (stem and root). The stage rotated 180°, with images collected every 0.1° with 600 ms or 300 ms exposure time for a total scan time of ~18 min. Paired stem and root scans were measured in quick succession with <5 min taken to reposition the sample and stage.

### Image reconstruction analysis and vulnerability curve construction

Acquired 2D longitudinal raw images were reconstructed into a series of 2159 cross-sectional images with XLICT workflow 2015 (CSIRO) using the FBP (Paganin *et al.*, 2002) reconstruction algorithm. For each scan, a cross-sectional slice was selected for analysis based on clarity. When plants were scanned at multiple time points, the precise scan location was marked with correction fluid on the stem and root, and slices used for analysis came from the same position within the scan zone. Embolized vessels were measured (area and diameter) and counted using ‘Threshold’ and ‘Analyze particles’ functions in Image J software (1.48). Total vessel counts were performed manually on fully embolized ‘cut’ scans in most cases. For *E. saligna*, total vessel counts were obtained from scans where both air- and water-filled vessels were clear and easy to distinguish. Vulnerability curves for *C. deodara* were based on measurement of embolized xylem cross-sectional area, since the spatial resolution of images attained at IMBL was not sufficient to count individual tracheids in this species.

We used the percentage of embolized conduits to estimate hydraulic impairment during drought. Using the percentage of embolized conduits could potentially underestimate vulnerability for species with a wide distribution of vessel diameters (Brodersen *et al.*, 2013); however, our data demonstrate that this issue does not significantly impact the shape of vulnerability curves or estimates of vulnerability curve parameters in the majority of species (Gauthey *et al.*, 2020; Li *et al.*, 2020). Therefore, the percentage of embolized conduits was plotted against water potential for all samples. A re-parameterized Weibull function was fitted to produce a vulnerability curve. From the curve, we calculated the stem xylem water potential (xylem tension) at which 12% and 50% of vessels were embolized (P_12_ and P_50_, respectively) and 95% confidence intervals (CIs) using the *fitplc* package (Duursma and Choat, 2017) in R v.3.2.0 (R Core Team, 2015). Statistically significant differences between roots and stem P_50_ were assessed for each species by generating 95% CIs for the difference between group means (Gauthey *et al.*, 2020). We resampled 2000 values from bootstrap distributions generated during curve fitting in *fitplc* and used them to generate bootstrap CIs for the difference between mean P_50_ for roots and stems. Significant differences were assessed by overlap of these CIs with zero.

### Embolism spread

Two-dimensional cross-sectional images were examined for patterns of embolism spread as xylem water potential decreased. Individual plants were rescanned at the same location, making patterns of embolism propagation easy to follow. By determining the location and distribution of embolized conduits over the course of the dehydration, a general pattern of spread could be defined for each species. Native embolism present in xylem conduits at the start of the experiment under well-hydrated conditions was defined as the mean percentage embolism from initial scans of each plant if the first scan occurred at a water potential higher than the calculated P_12_ for that species. Three-dimensional volume rendering and segmentation of embolized conduits was performed with the open-source visualization software Drishti (Limaye, 2012).

### Synthesis of literature data

A dataset was compiled from the Xylem Functional Traits database (Choat *et al.*, 2012), updated with studies published between 2015 and 2018. The dataset included xylem vulnerability to cavitation (P_50_) data for roots and stems of 87 species and 131 distinct populations, comprising 67 angiosperms and 64 conifers (Supplementary Table S1). Data were drawn from 36 studies in which roots and stems were measured on the same plants. We analysed the relationship between root and stem vulnerability to cavitation for angiosperm and conifer species given the fundamental differences in xylem anatomy between these groups. Data for angiosperms were also separated into diffuse porous, ring porous, and semi-ring porous categories to examine the effect of xylem anatomy on estimates of vulnerability to cavitation in roots and stems. We calculated the ratio of stem to root P_50_ for each record in the database. To overcome non-normal distributions, unequal variances, and large differences in sample size, we used the conservative, non-parametric Mann–Whitney–Wilcoxon test to assess the probability that the vulnerability ratios generated in this study and those generated using imaging techniques together are statistically similar populations from the historical dataset.

## Results

### Vulnerability to cavitation

**Fig. 1.**
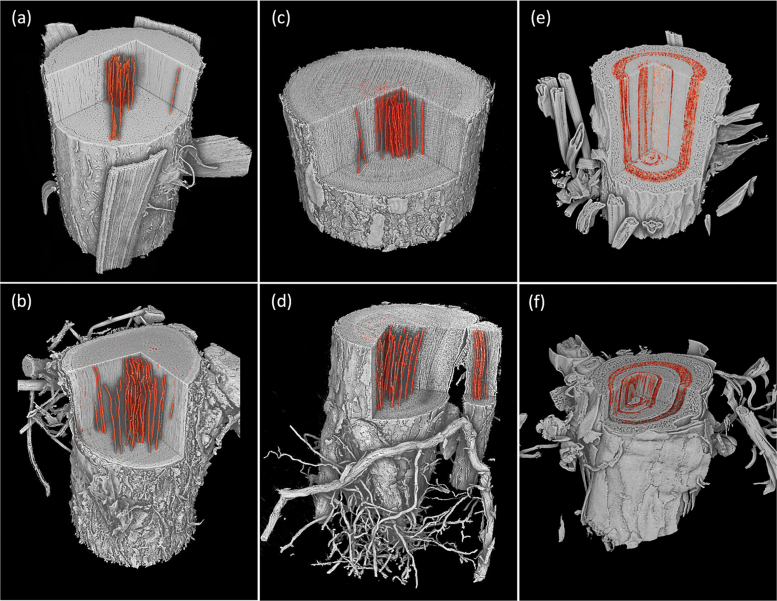
Representative images showing rendering of stem and root volumes visualized using X-ray microtomography from one individual of *Acacia aneura* (A and B), *Eucalyptus saligna* (C and D), and *Cedrus deodara* (E and F). Cut away in root and stem volumes shows the internal anatomy of the plant with embolized vessels or tracheids highlighted in orange. Cut away sections in root and stem volumes show the internal anatomy of the plant, with embolized vessels rendered in orange for angiosperm species *A. aneura* and *E. saligna*. In stems and roots of the conifer species *C. deodara*, the area of embolized xylem tracheids is shown in orange. The field of view for scans was 28 mm in *X* and *Y* orientations and 20 mm in *Z* (vertical) orientation for stems and roots. A sub-volume of scans has been rendered in each panel. (This figure is available in colour at JXB online.)

**Fig. 2.**
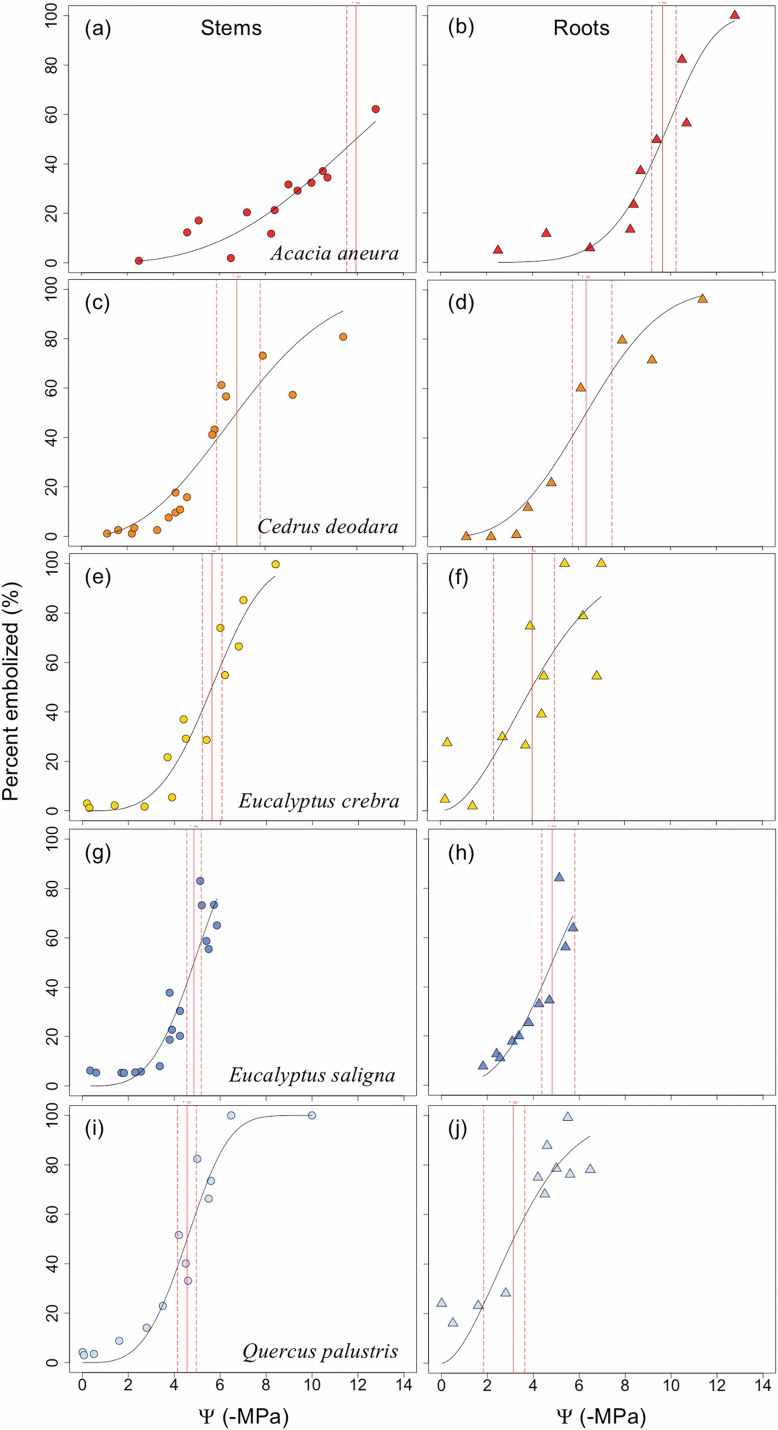
Xylem vulnerability to cavitation curves for stems (circles) and roots (triangles) of five species, *Acacia aneura* (A and B), *Cedrus deodara* (C and D), *Eucalyptus crebra* (E and F), *Eucalyptus saligna* (G and H), and *Quercus palustris* (I and J). Data are derived from counts of embolized conduits observed in microCT images during dehydration. The water potential at which 50% of conduits become embolized (P50) is indicated by a vertical red line, with dashed lines showing 95% confidence intervals. (This figure is available in colour at JXB online.)

**Fig. 3.**
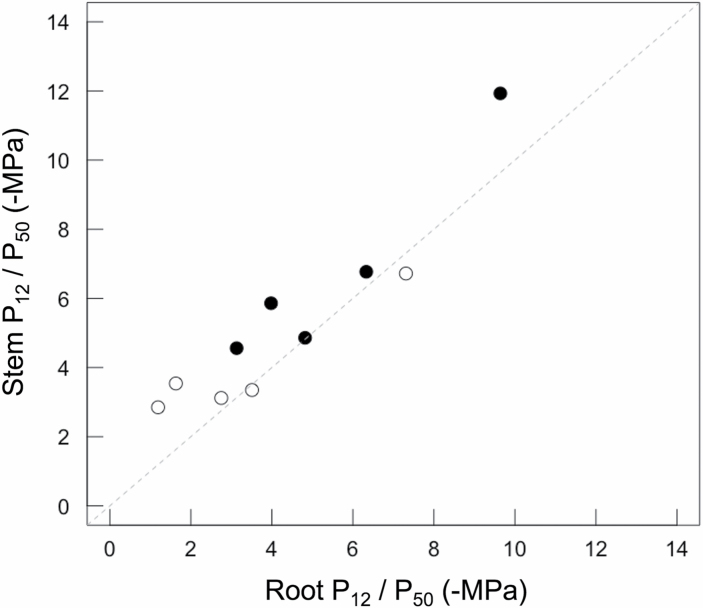
Scatterplot showing the relationship between estimates of P50 (filled symbols) and P12 (open symbols) for roots and stems of five woody species. Estimates of P50 and P12 were derived from vulnerability curves generated from microCT imaging of dehydrating, intact plants. Across species, P50 values of roots and stems were significantly related (regression slope=1.17; *r*2=0.91; *P*=0.01). Values of P12 were also related in stems and roots (regression slope=0.65; *r*2=0.87; *P*=0.02). Lines were fit with a standardized major axis model and did not differ from the 1:1 relationship (dashed line) between vulnerability parameters.

MicroCT images of intact plants revealed the spatial patterns of embolism formation and spread in the xylem tissue of stems and roots of five woody species (Fig. 1). Vulnerability to cavitation, defined as the water potential causing a 50% loss of hydraulic function (P_50_), varied broadly among the selected species, ranging from –4.51 MPa in stems of *Quercus palustris* to –11.93 MPa in stems of *Acacia aneura* (Table 1; Fig. 2); P_50_ in roots varied from –3.13 MPa in *Q. palustris* to –9.64 MPa in *A. aneura* (Table 1; Fig. 2). Across species, P_50_ values of roots and stems were significantly related (regression slope=1.12; *r*^2^=0.88; *P*=0.01), meaning that species with more vulnerable stems also had more vulnerable roots (Fig. 3). The point of incipient cavitation (P_12_) was also strongly related in stems and roots (regression slope=0.61; *r*^2^=0.83; *P*=0.02). While there was strong convergence in root and stem vulnerability to cavitation, the P_50_ of roots was significantly higher than the P_50_ of stems in three species (*A. aneura*, *E. crebra*, and *Q. palustris*); the P_50_ of roots and stems was not significantly different in the other two species (*E. saligna* and *C. deodara*) (Table 1; Supplementary Fig. S2). P_12_ was not significantly different for roots and shoots in four of the five species, while, in *Q. palustris*, root P_12_ was higher (less negative) although this is likely to have been caused by the presence of native embolism in the samples (Table 1). The difference between root and stem P_50_ for the five species was small, ranging from 1% to 31% of the stem P_50_. While it was possible to extract estimates of P_88_ from curve fits, these values were based on extrapolation beyond the range of measured data for some species. A test of statistical significance between root and stem P_88_ was not possible because upper CIs could not be calculated.

### Patterns of embolism spread

Observations indicated that primary xylem vessels in the stems directly adjacent to the pith were generally embolized even in well-watered plants and as such were excluded from analysis. No differentiation was made between primary and secondary xylem in the roots, as primary xylem was not resolvable. Native embolism was <10% in stems of all species and in roots of *A. aneura*, *C. deodara*, and *E. saligna* (Table 1). Some roots of *E. crebra* and *Q. palustris* contained higher levels of native embolism, with means of 11.3% and 21.1%, respectively (Table 1). Native embolism in stems was primarily confined to the central third of the xylem area, with a few isolated embolized conduits outside this area for all species.

**Fig. 4.**
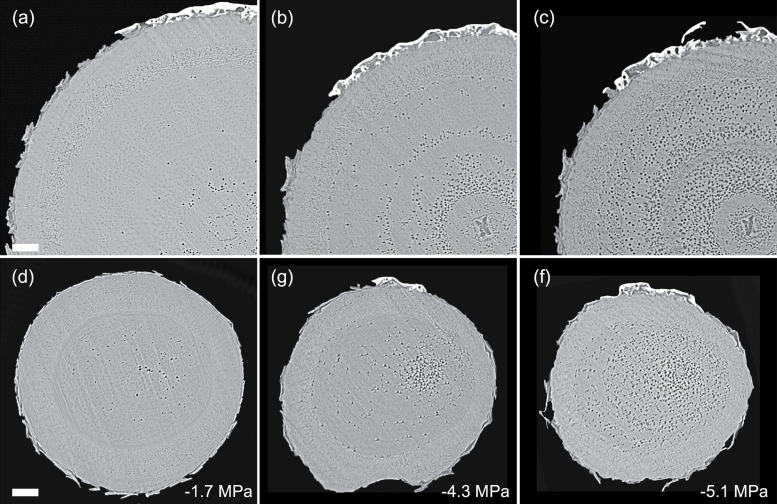
Transverse slices from microCT scans showing stems (A–C) and roots (D–F) of *Eucalyptus saligna*. Images are taken from a time series of scans on a single plant during dehydration treatment. The number of embolized vessels (black) increases with decreasing xylem water potential. Scale bars are equal to 1000 μm in all panels.

**Fig. 5.**
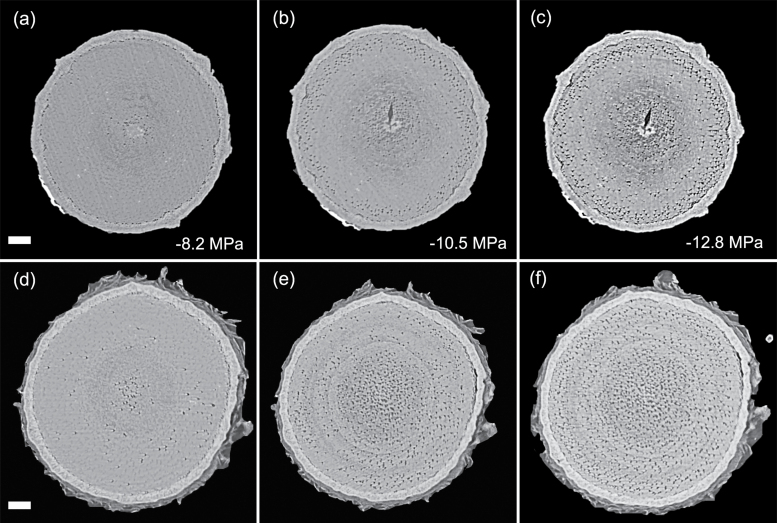
Transverse slices from microCT scans showing stems (A–C) and roots (D–F) of *Acacia aneura*. Images are taken from a time series of scans on individual during a dehydration treatment. The number of embolized vessels (black) increases with decreasing xylem water potential. Water potentials (MPa) are shown at the time of each scan. Scale bars are equal to 1000 μm in all panels.

**Fig. 6.**
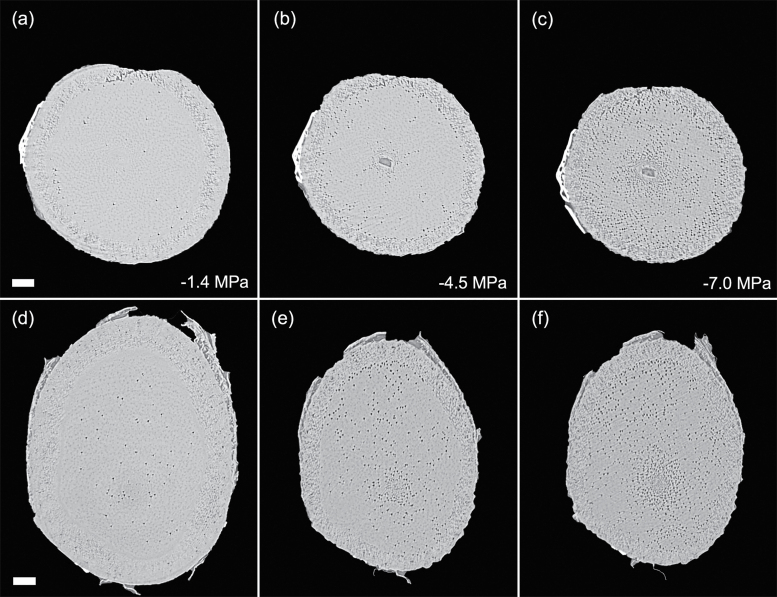
Transverse slices from microCT scans showing stems (A–C) and roots (D–F) of *Eucalyptus crebra*. Images are taken from a time series of scans on a single plant during dehydration treatment. The number of embolized vessels (black) increases with decreasing xylem water potential. Water potentials (MPa) are shown at the time of each scan. Scale bars are equal to 1000 μm in all panels.

**Fig. 7.**
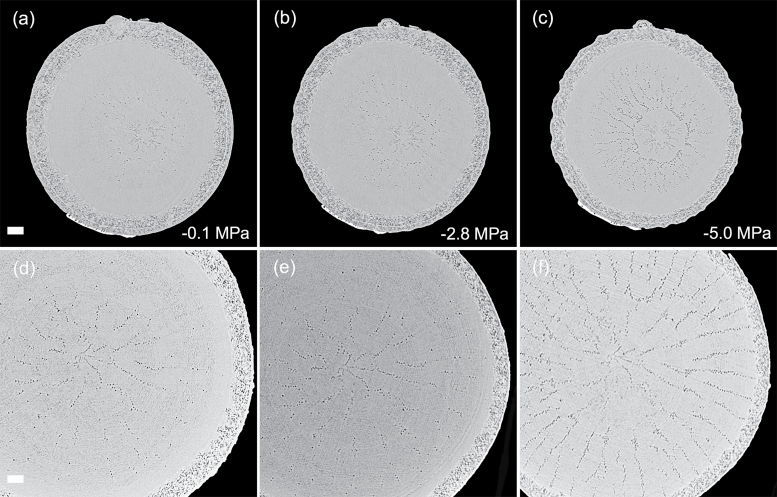
Transverse slices from microCT scans showing stems (A–C) and roots (D–F) of *Quercus palustris*. Images are taken from a time series of scans on a single plant during dehydration treatment. The number of embolized vessels (black) increases with decreasing xylem water potential. Water potentials (MPa) are shown at the time of each scan. Scale bars are equal to 1000 μm in all panels.

**Fig. 8.**
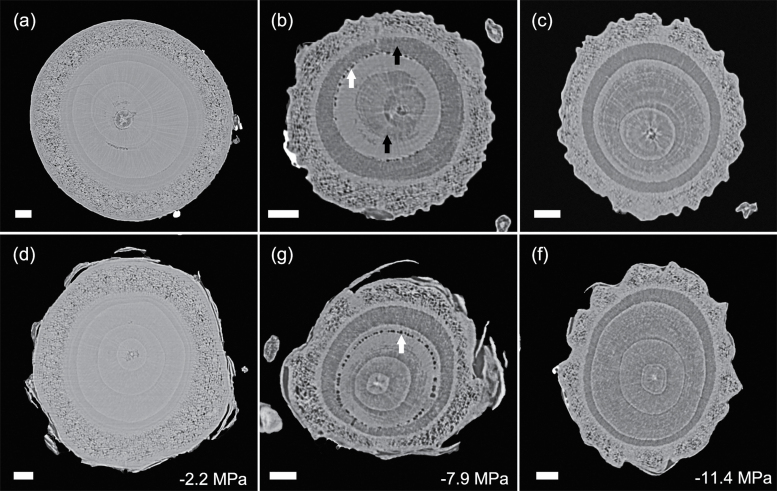
Transverse slices from microCT scans showing stems (A–C) and roots (D–F) of *Cedrus deodara*. Images are taken from a time series of scans during a dehydration treatment. Each vertical pair is a concurrent stem and root scan for one individual. Individuals differed between time points. The area of embolized tracheid (black arrows in B) increased with decreasing xylem water potential. Water-filled tracheids appear light grey. Empty resin canals (white arrows in B and G) can be seen in some slices. Water potentials (MPa) are shown at the time of each scan. Scale bars are equal to 1000 μm in all panels.

Patterns of embolism spread varied between species and organ as plants dehydrated. In the angiosperm species, some embolized vessels were generally present in the centre of the stem close to the pith at the initial scan time point (Figs 4–7). Embolism spread outwards towards the cambium as plants dehydrated and Ψ _X_ declined. Although embolism usually spread between neighbouring conduits, suggesting air seeding between connected vessels, there were many instances in which embolism appeared to ‘jump’ between different sections of the stem. For instance, in stems of *E. saligna*, embolism appeared to spread radially within weakly defined seasonal growth rings (Fig. 4). The spatial pattern of embolized vessels was less clear in the roots of angiosperm species. Vessels near the centre did appear to embolize earlier during dehydration, but overall embolism was much more evenly distributed in roots compared with stems. In the stems and roots of the conifer species *C. deodara*, large blocks of tracheids often cavitated in one event, with the outer growth ring becoming embolized prior to the inner rings (Fig. 8).

**Fig. 9.**
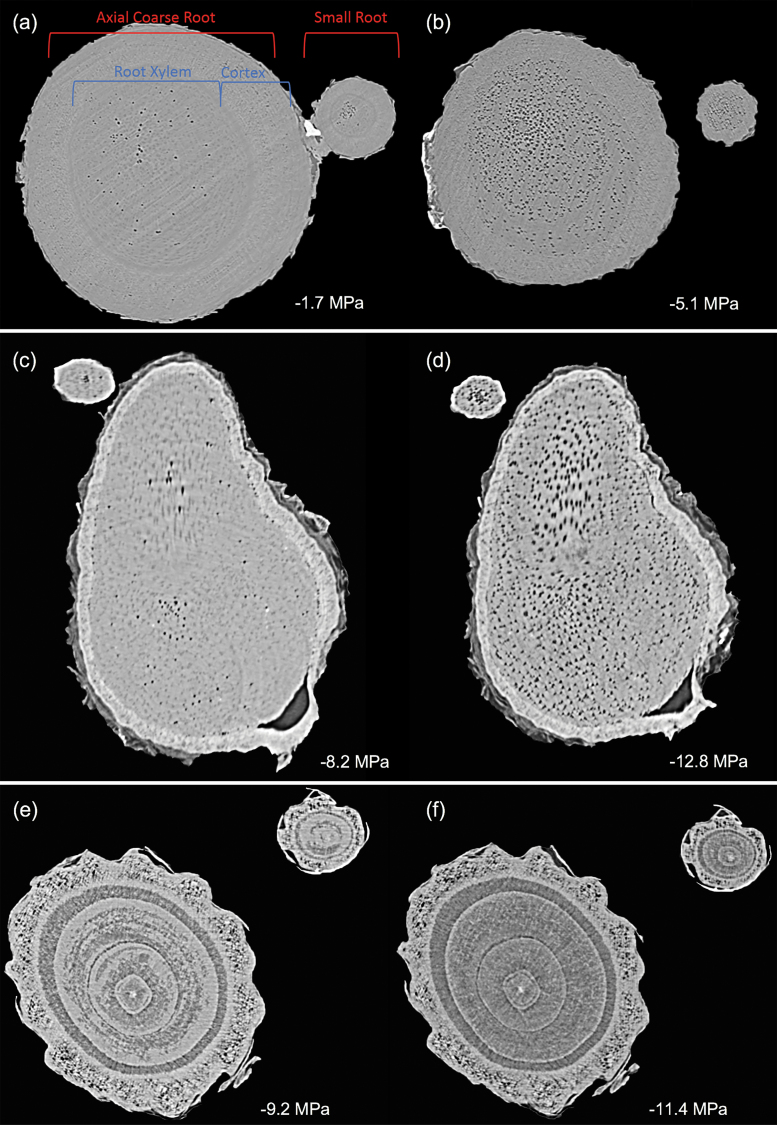
Transverse slices showing embolism in a small root and the main root at initial and final scan points for one individual of three species: *Eucalyptus saligna* (A and B) *Acacia aneura* (C and D), and *Cedrus deodara* (E and F). Water potentials (MPa) are shown at the time of each scan.

While the target of this experiment was to capture coarse axial roots, smaller adjacent roots were repeatedly imaged throughout the dehydration period in some individuals of *E. saligna*, *A. aneura*, and *C. deodara* (Fig. 9). The image resolution precluded counts of total vessel number of these smaller roots, but accumulation of embolism appeared proportional in the small root compared with the larger root.

### Analysis of literature data

**Fig. 10.**
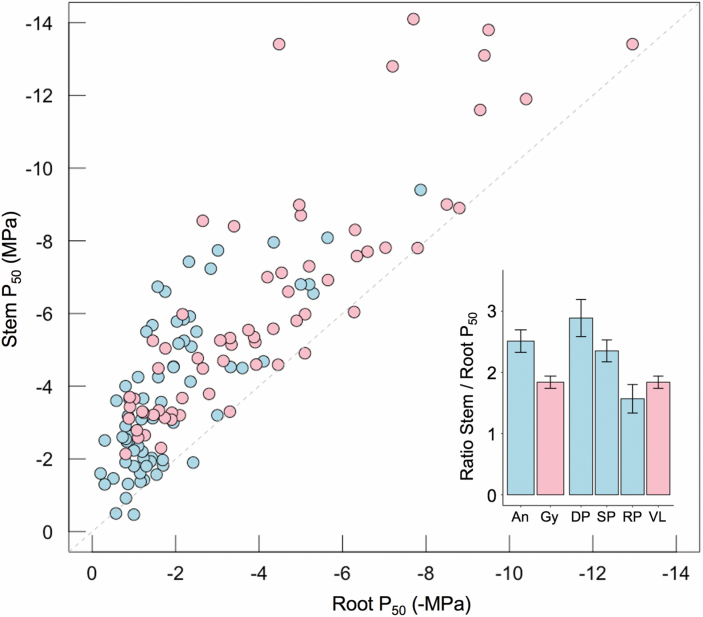
Data synthesis showing the relationship between stem and root P50 values based on previously published studies. Data are shown for a total of 87 species with 67 independent populations for angiosperms (blue) and 64 populations for conifers (pink) measured in studies published between 1994 and 2018. The dashed line shows the 1:1 relationship. Inset shows the ratio of the stem to root P50 within a plant as a function of group (An, angiosperm; Gy, gymnosperm) and xylem anatomy (DP, diffuse porous; SP, semi-ring porous; RP, ring porous, VL, vesselless) with bars showing the SE. Data and references used in this analysis are provided inSupplementary Table S1. (This figure is available in colour at JXB online.)

Between 1994 and 2018, 36 studies compared root and stem hydraulic vulnerability across 87 species including 131 populations (Fig. 10). Within the dataset, ~94% of gymnosperms and 96% of angiosperms were reported to have roots with xylem that was more vulnerable to cavitation than stems of the same plant. On average, angiosperm roots were 2.5-fold more vulnerable than stems within a plant, while conifer roots were ~1.7 times more vulnerable than stems. Angiosperm species with diffuse porous xylem structure showed the greatest divergence between the root and stem P_50_ (Fig. 10). As a group, conifers (vesselless) exhibited less difference between root and stem P_50_ than angiosperms. When examining results as a function of the method used to generate P_50_, it was clear that centrifuge and air injection methods resulted in a greater divergence of root and stem P_50_ than bench dehydration and visual methods (Supplementary Fig. S3). This difference was greatest for centrifuge measurements applied to angiosperm species. We found that datasets containing our data alone (*n*=5) and all data thus far generated by visual methods (*n*=9) were statistically distinct from data generated using traditional methods (*P*=0.03 and *P*=0.002, respectively).

## Discussion

In this study we utilized microCT imaging to non-invasively evaluate the drought-induced vulnerability to cavitation of roots and stems in five woody species. Imaging of intact plants exposed to drought stress revealed a strong convergence between root and stem P_50_ across species. Within a plant, P_50_ was significantly higher (less negative) in roots of three species, while values of P_12_ were not significantly different for the majority of the species (Table 1). While our findings suggest that roots are more vulnerable to cavitation than stems in some species, the differences in P_50_ were not as great as those observed in previous studies. For the majority of species examined here, roots would not be expected to reach P_50_ before any significant cavitation had occurred in the stem. Results of this nature are critical for parameterization of process-based models seeking to predict the response of plants to drought, particularly those focused on simulation of plant mortality.

### Vulnerability to cavitation of roots and stems

Studies published over the last three decades indicate that, within individuals, woody roots are often less resistant to cavitation than stems, sometimes dramatically so (Sperry and Saliendra, 1994; McElrone *et al.*, 2004; Maherali *et al.*, 2006; Pratt *et al.*, 2007; Bartlett *et al.*, 2016). This body of work suggests that catastrophic levels of cavitation may occur in woody roots well before cavitation thresholds are reached in stems. It is true that roots should experience the least negative water potential values in the hydraulic pathway while the plant is transpiring and that this would reduce the probability of cavitation occurring in the roots during mild water stress. However, during a severe drought, stomata close and roots may decouple from the soil, leading to a collapse of water potential gradients along the hydraulic pathway (Cuneo *et al.*, 2016; Choat *et al.*, 2018). In this situation, xylem water potentials of woody stems and roots would be close to equilibrium, leaving cavitation thresholds as the major determinant of where significant cavitation would occur first. Thus, if roots were significantly less resistant to cavitation than stems, hydraulic failure at the whole-plant level would be dictated by cavitation in the roots.

Our results indicated that the vulnerability of roots was not dramatically different from that of stems (Fig. 2). In two of the species that exhibited greater vulnerability in roots, *E. crebra* and *Q. palustris*, higher levels of native embolism were present in roots than in stems (Table 1). This native embolism is likely to have influenced some vulnerability curve parameters, particularly P_12_ and P_50_. It is notable that *Q. palustris* was the only species in which P_12_ was significantly higher in roots compared with stems. The significantly higher values of P_12_ and P_50_ for *Q. palustris* roots could be viewed as an artefact caused by native embolism given the impact on curve fit and shape. While it is possible that some native embolism may have been induced when soil was removed from the roots in *Q. palustris*, this is unlikely given the thickness of roots being imaged and lack of native embolism in roots of other species exposed to the same treatments (Table 1). The most likely explanation for the presence of higher levels of native embolism in roots of intact plants is greater vulnerability to cavitation compared with stems. Regardless, our results suggest that within-plant differences in root and stem vulnerability are not as large as previously observed for many species.

Our findings are therefore consistent with the small number of studies in which vulnerability was evaluated in roots by non-invasive or *in situ* imaging techniques (Cuneo *et al.*, 2016; Skelton *et al.*, 2017*a*; Rodriguez-Dominguez *et al.*, 2018; Losso *et al.*, 2019); these studies showed small or no significant difference in vulnerability between roots and stems within a plant. Our dataset doubles the number of species in which roots and shoots are compared with non-invasive imaging techniques and extends the results to larger plants and a greater range of xylem functional types. Together, these data suggest that root xylem may be more resistant to cavitation than previously thought. Further, they also suggest that vulnerability in stems and roots is coordinated such that the function of one organ is not compromised significantly before the other during severe drought (Rodriguez-Dominguez *et al.*, 2018). This coordination would increase the probability of survival under extreme drought while allowing for more rapid recovery after water stress was relieved by significant precipitation (Skelton *et al.*, 2017*b*; Creek *et al.*, 2018).

Whether the discrepancy between previous findings and those based on non-invasive imaging is the result of methodological error or variation in other factors (species or developmental stage) is difficult to determine at present. The current study greatly increased the age and size of plant material used in imaging studies, fully leveraging the available X-ray hutch space. However, imaging methods are still restricted to relatively young, potted plants if intact plant material is to be visualized. In contrast, the majority of previous vulnerability studies have used branches and roots harvested from mature trees. The impact of life stage on xylem vulnerability is likely to differ by species, with some research finding only slight variations between saplings and mature trees (Wason *et al.*, 2018) and others finding larger differences (Domec and Gartner, 2003).

It is also possible that vulnerability has been overestimated in the roots of angiosperm species due to methodical artefacts associated with two commonly used hydraulic techniques, air injection and centrifugation (Cochard *et al.*, 2013; Jansen *et al.*, 2015). Almost all data for root vulnerability have been generated using these techniques and the artefact is contingent on the proportion of open vessels in the segment used for measurement (Cochard *et al.*, 2010; Ennajeh *et al.*, 2011; López *et al.*, 2018). As such, roots may be particularly prone because of their generally longer and wider vessels compared with stem xylem tissue (Zimmermann and Potter, 1982; Kolb and Sperry, 1999). This could lead to large differences in the P_50_ of roots and stems that do not reflect vulnerability of these organs in intact plants. Evidence for this is provided by the greater divergence in root and stem vulnerability of angiosperms compared with conifers (Fig. 10). This is particularly true for diffuse porous angiosperms, in which root xylem vessels may exceed the length threshold (Fig. 10 inset). This contrasts with ring porous species in which both stems and roots would be expected to exceed length thresholds, leading to smaller differences in root and stem vulnerability. While the generality of these artefacts has been debated (Pratt *et al.*, 2015), the uncertainty relating to root xylem parameters warrants further investigation given their importance to whole-plant function.

Although our results indicate that vulnerability is largely conserved between stems and coarse woody roots, it may also vary across the root system (McElrone *et al.*, 2004). Sperry and Ikeda (1997) observed differences in vulnerability between root orders, with small woody roots being more vulnerable than larger roots. While we were not able to quantify levels of embolism in the lower orders of roots, it was possible to observe trends in embolism formation and thus make qualitative comparisons with larger roots. Smaller roots appeared to follow the same general pattern of embolism formation as adjacent larger roots (Fig. 9). However, we were not able to resolve the individual conduits or the cortex of fine roots, which are more vulnerable to cavitation than coarse roots in *Vitis vinifera* (Cuneo *et al.*, 2016). This may reflect vulnerability segmentation of distal organs, with fine roots continuously turned over while coarse roots are maintained for longer periods since they represent a larger carbon cost to the plant (Tyree and Zimmermann, 2002). Further work is necessary to establish this pattern more generally.

Loss of conductance can also occur outside the xylem via a range of mechanisms including changes in aquaporin expression (McElrone *et al.*, 2007; Maurel *et al.*, 2008; Gambetta *et al.*, 2012), formation of cortical lacunae in fine roots (Cuneo *et al.*, 2016), and disconnection of roots from the soil (Nobel and Cui, 1992; Carminati *et al.*, 2009). However, these mechanisms are likely to be transitory, with rapid recovery of hydraulic conductance facilitated by rewetting of soils, up-regulation of aquaporin expression, and production of new fine roots. In the present study, we focused on vulnerability to cavitation in woody stems and roots, a trait that has been quantitively linked to mortality induced by water stress (Brodribb and Cochard, 2009; Urli *et al.*, 2013; Hammond *et al.*, 2019). Future work examining how vulnerability varies across root orders and outside of the xylem is highly desirable.

### Spatial patterns of embolism spread

Perhaps the greatest advantage of using microCT imaging to examine xylem response to drought is the spatial and temporal information it provides. Such data may inform our understanding of the mechanisms involved in cavitation and help link loss in conductivity to anatomical features within the xylem tissues. Analysis of embolism spread within stem and root cross-sections revealed that some level of native embolism was present, even at high water status (Figs 4–8). This minimal native embolism occurred as clusters of vessels that were generally located in the older xylem (closer to the pith). These clusters are presumably the result of previous stress events during the early growth stages of the plant. There were also occurrences of isolated embolized vessels that were apparently not connected to other gas-filled vessels. Such isolated embolized conduits have been observed in other microCT studies covering a range of xylem functional groups including conifers, ring porous, and diffuse porous species (Brodersen *et al.*, 2013; Knipfer *et al.*, 2015; Choat *et al.*, 2016). It remains unknown how these isolated conduits become embolized, although it may relate to defects in the conduit walls when they were initially formed or gas entry from surrounding tissue (e.g. intercellular air spaces or fibres).

There was little change in embolism during dehydration until a critical water potential was reached, after which the levels of embolism increased steeply in stems and roots of all species. In stems, embolism generally spread from the centre outwards. This spread of embolism presumably occurred by air seeding across pit membranes of adjacent vessels and tracheids (Tyree and Sperry, 1989; Cochard *et al.*, 1992; Brodersen *et al.*, 2013). As more extreme water potentials were reached, embolism spread across the remainder of the cross-section. Patterns of embolism spread differed between species. In *E. saligna*, radial spread of embolism in stems followed weakly differentiated seasonal growth rings, with embolism spreading preferentially around the interior vessels of these rings (Fig. 4). In *C. deodara*, embolism was initially present only in a small number of tracheids adjacent to the pith (Fig. 8A, D). With declining Ψ _x_, embolism occurred in large blocks of tracheids in the outermost growth ring (Fig. 8B, E). In some cases, the entire current year growth ring became embolized between two scan points, suggesting a single large cavitation event. This is consistent with observations from previous studies involving visualization of embolism in conifer species (Choat *et al.*, 2015; Brodribb *et al.*, 2016) and suggests that there is a very low variation in air seeding thresholds within the growth ring. Patterns of embolism spread were very similar between stems and roots in *C. deodara*. In angiosperm species, the spread of embolism occurred in a more random fashion in roots, although embolism generally spread from the inner sections of the xylem towards the cambium.

### Evaluation of xylem function by microCT

While non-invasive imaging techniques provide some clear advantages over destructive methodology, recent studies have raised doubts over the reliability of microCT based on damage to living cells caused by high X-ray doses delivered to plants during scanning (Savi *et al.*, 2017; Petruzzellis *et al.*, 2018). These concerns are mainly related to the study of embolism repair, which putatively relies on living cells in the xylem. Although high X-ray doses will undoubtedly cause damage to living cells, we note that Brodersen *et al.* (2010) were able to observe refilling in grapevines that were repeatedly scanned over a 24 h period. This suggests that damage done to living cells by X-rays does not alter physiological processes within short time scales (hours to days) but may be important to consider over longer time frames (weeks to months). Further work is required to fully understand the impacts of X-ray radiation on living cells in the xylem and the implications of using this technique to study embolism repair.

Concerns have also been raised over the use of microCT to measure vulnerability to cavitation based on radiation dose (Petruzzellis *et al.*, 2018) and the challenges associated with translating anatomical images into loss of hydraulic function (Venturas *et al.*, 2019). However, vulnerability curves generated by microCT have now been validated independently by a number of studies and are generally in close agreement with standard hydraulic techniques (Brodersen *et al.*, 2013; Torres-Ruiz *et al.*, 2014; Choat *et al.*, 2016; Nardini *et al.*, 2017; Nolf *et al.*, 2017; Gauthey *et al.*, 2020). These studies provide convincing evidence that microCT can accurately predict the impacts of embolism on hydraulic conductance although it is important to consider potential artefacts. Based on validation studies, it does not appear that the X-ray radiation exposure alters vulnerability curves. There is no evidence that X-ray radiation causes cavitation during microCT image acquisition; repeated scans of samples at the same xylem tension do not yield new embolism (Choat *et al.*, 2016; Venturas *et al.*, 2019). Error in estimating the level of hydraulic impairment may also be caused by the occurrence of non-functional or hydraulically isolated xylem vessels, which may remain water filled in 2D cross-sectional images during dehydration (Nolf *et al.*, 2017; Choat *et al.*, 2019). This phenomenon has the potential to cause underestimation of vulnerability based on analysis of water-filled and gas-filled vessels. Once again, previous validation studies suggest that these issues do not cause significant divergence between microCT- and hydraulic-based vulnerability curves. There is also a possibility that divergence between microCT and hydraulic estimates of vulnerability could be caused by artefacts associated with hydraulic measurements, as there are numerous sources of experimental error associated with measurement of flow rates through the xylem (Espino and Schenk, 2011; Jansen *et al.*, 2015; De Baerdemaeker *et al.*, 2019).

### Conclusions

Our results are consistent with previous studies utilizing non-invasive and *in situ* imaging techniques. However, they contrast with the majority of previous literature using other methodologies and the broadly held view that roots are dramatically less resistant to cavitation than stems (Alder *et al.*, 1996; Hacke and Sauter, 1996; Sperry and Ikeda, 1997; McElrone *et al.*, 2004; Pratt *et al.*, 2015; Johnson *et al.*, 2016). Providing greater certainty in this area is critical for process-based models that utilize hydraulic threshold values to predict drought-induced mortality (Mackay *et al.*, 2015; Johnson *et al.*, 2018). It is clear that more work is required to establish general trends in the coordination of hydraulic thresholds between roots and shoots. Studies utilizing non-invasive imaging methods are often constrained by access to facilities, beam time, and hutch space. As such, replication across species with these methods has thus far been limited. It will also be important to integrate experiments examining loss of conductance in the root system that occurs outside of the xylem components of the hydraulic pathway and to investigate variation across root orders (Cuneo *et al.*, 2016). Determining how such losses of conductance relate to cavitation thresholds in coarse woody roots will be essential for a full understanding of plant hydraulic response to drought.

## Supplementary data

The following supplementary data are available at *JXB* online.

Table S1. Dataset from previously published studies used to compare vulnerability to cavitation in roots and stems, including method and wood porosity type.

Fig. S1. Correlation between stem water potential measured with stem psychrometers and covered leaves in a pressure chamber for three species

Fig. S2. Difference of means and 95% confidence intervals for root and stem P_50_.

Fig. S3. Data synthesis from previously published studies showing the relationship between stem and root P_50_ values.

eraa381_suppl_Supplementary_File001

## Data Availability

All data supporting the findings of this study are available within the paper and within its supplementary data published online.
