# Supplementary material for: Non-invasive imaging reveals convergence in root and stem vulnerability to cavitation across five tree species
Source: J Exp Bot. 2020 Sep 18;71(20):6623–37. doi: 10.1093/jxb/eraa381 (PMC7586747; doi:10.1093/jxb/eraa381)
Supplement: eraa381_suppl_Supplementary_File001 [file eraa381_suppl_supplementary_file001.pdf]

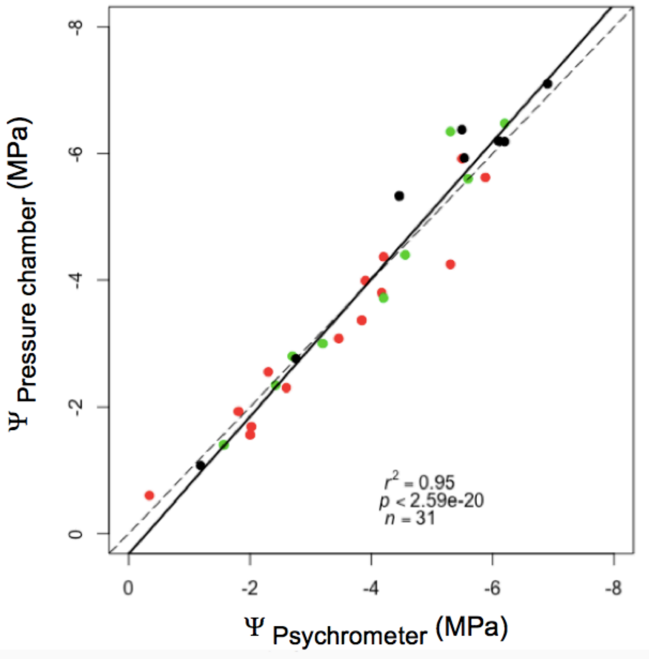

Supplementary Figure S1. Correlation between stem water potential measured with stems psychrometers and covered leaves in a pressure chamber for three species, *Eucalyptus saligna* (black circles), *Eucalyptus crebra* (black circles), *Quercus palustis* (green circles), during experimental dehydration. A standardized major axis model fit to pooled dataset (dashed line) did not differ from the 1:1 relationship (solid line).

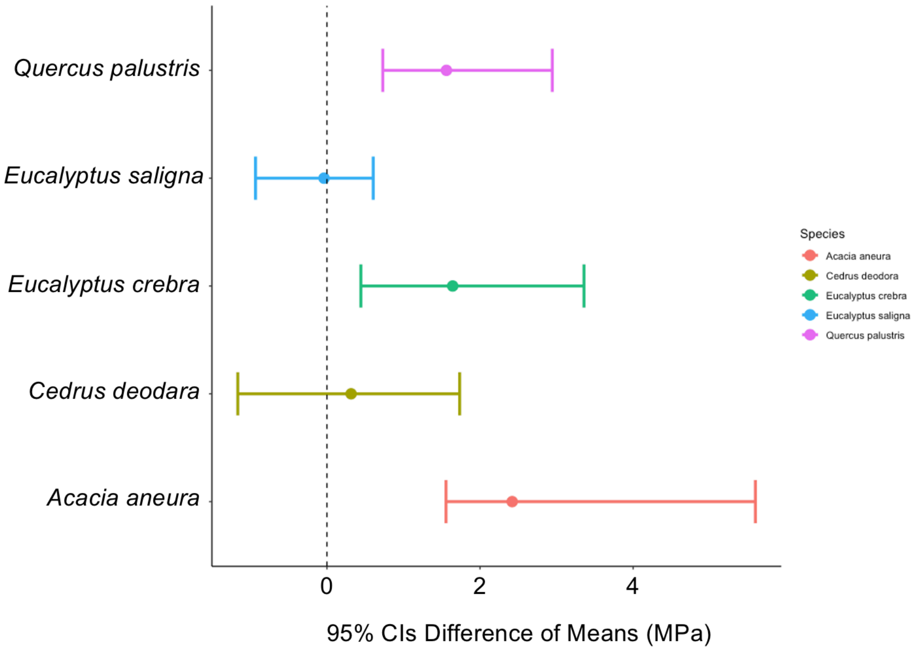

Figure S2. Difference of means for root and shoot P50 of five woody plant species. Bars show 95% confidence intervals for difference of means. Where 95% CIs overlap with zero, there is no significant difference between root and stem P50 for that species.

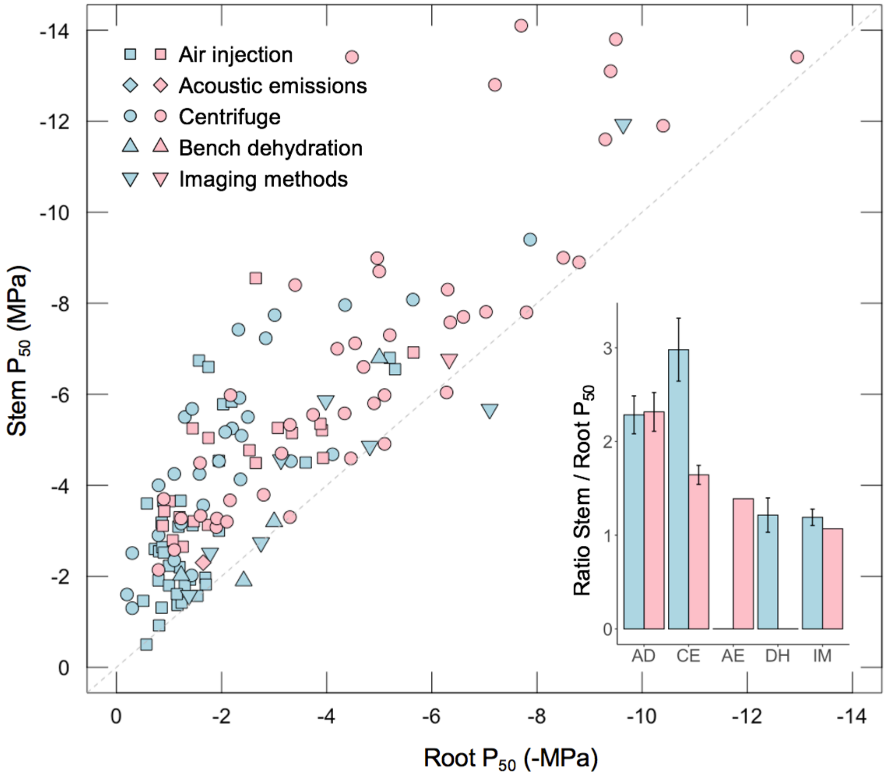

Figure S3. Data synthesis showing the relationship between stem and root P_{50} values based on previously published studies. Data are shown for a total of 87 species with 67 independent populations for angiosperms (blue) and 64 populations for conifers (pink) measured in studies published between 1994 and 2018. Within each group, methodology used to measure vulnerability is shown by symbol. The dashed line shows the 1:1 relationship. Inset shows the ratio stem to root P_{50} within a plant as a function of methodology (AD = air injection, CE = centrifuge, AE = acoustic emissions, DH = bench dehydration, IM = imaging methods). Data and references used in this analysis are provided in Table S1.

Table S1. Values of water potential at 50% loss of hydraulic conductivity (P_{50}) for roots and stems, including method (DH = dehydration, CE = centrifuge, AD = air-injection, AE = acoustic emissions, IM=imaging) and wood porosity type (diffuse-porous, semi-ring-porous, ring porous, vesseless). Data were taken from the Xylem Functional Traits database (Choat et al. 2012), updated from papers published between 2012-present.

Group	Species	Root P_{50} (MPa)	Stem P_{50} (MPa)	Method	Porosity	Reference
Angiosperm	*Acacia aneura*	-9.64	-11.93	IM	diffuse-porous	Peters et al. in review
Angiosperm	*Acer grandidentatum*	-1.22	-3.66	AD	diffuse-porous	Alder et al. 1996
Angiosperm	*Acer grandidentatum*	-0.58	-3.60	AD	diffuse-porous	Alder et al. 1996
Angiosperm	*Acer grandidentatum*	-0.80	-4.00	CE	diffuse-porous	Sperry & Hacke 2004
Angiosperm	*Acer monspessulanum*	-1.57	-6.74	AD	diffuse-porous	Martinez-Vilalta et al. 2002
Angiosperm	*Acer pseudoplatanus*	-1.78	-2.51	IM	diffuse-porous	Losso et al. 2019
Angiosperm	*Acer rubrum*	-1.69	-1.97	AD	diffuse-porous	Maherali et al. 2006
Angiosperm	*Acer saccharum*	-2.42	-1.90	DH	diffuse-porous	Tsuda & Tyree 1997
Angiosperm	*Alnus glutinosa*	-1.20	-2.20	AD	diffuse-porous	Hacke & Sauter 1996
Angiosperm	*Alnus incana*	-0.20	-1.60	CE	diffuse-porous	Hacke et al. 2001
Angiosperm	*Ambrosia dumosa*	-0.81	-2.55	AD	ring-porous	Mencuccini & Comstock 1997
Angiosperm	*Arbutus unedo*	-1.18	-3.09	AD	semi-ring-porous	Martinez-Vilalta et al. 2002
Angiosperm	*Atriplex canescens*	-1.30	-5.50	CE	diffuse-porous	Hacke et al. 2000a
Angiosperm	*Atriplex confertifolia*	-1.58	-4.25	CE	diffuse-porous	Sperry & Hacke 2002
Angiosperm	*Betula occidentalis*	-0.51	-1.46	AD	diffuse-porous	Sperry & Saliendra 1994
Angiosperm	*Betula occidentalis*	-1.10	-2.35	CE	diffuse-porous	Alder et al. 1997
Angiosperm	*Betula occidentalis*	-0.30	-1.30	CE	diffuse-porous	Hacke et al. 2001
Angiosperm	*Blepharocalyx salicifolius*	-1.40	-1.93	AD	diffuse-porous	Domec et al. 2006a
Angiosperm	*Ceanothus crassifolius*	-7.87	-9.40	CE	semi-ring-porous	Pratt et al. 2007a, b
Angiosperm	*Ceanothus crassifolius*	-2.84	-7.23	CE	semi-ring-porous	Pratt et al. 2008

Angiosperm	*Ceanothus cuneatus*	-4.35	-7.96	CE	semi-ring-porous	Pratt et al. 2007a, b
Angiosperm	*Ceanothus cuneatus*	-3.01	-7.74	CE	semi-ring-porous	Pratt et al. 2008
Angiosperm	*Ceanothus leucodermis*	-1.65	-3.56	CE	semi-ring-porous	Pratt et al. 2007a, b
Angiosperm	*Ceanothus leucodermis*	-3.32	-4.53	CE	semi-ring-porous	Pratt et al. 2008
Angiosperm	*Ceanothus megacarpus*	-5.64	-8.08	CE	semi-ring-porous	Pratt et al. 2007a, b
Angiosperm	*Ceanothus megacarpus*	-2.32	-7.42	CE	semi-ring-porous	Pratt et al. 2008
Angiosperm	*Ceanothus oliganthus*	-2.36	-4.13	CE	semi-ring-porous	Pratt et al. 2007a, b
Angiosperm	*Ceanothus oliganthus*	-1.95	-4.53	CE	semi-ring-porous	Pratt et al. 2008
Angiosperm	*Ceanothus spinosus*	-4.11	-4.68	CE	semi-ring-porous	Pratt et al. 2007a, b
Angiosperm	*Ceanothus spinosus*	-1.23	-3.16	CE	semi-ring-porous	Pratt et al. 2008
Angiosperm	*Cercis canadensis*	-0.90	-2.52	AD	semi-ring-porous	Maherali et al. 2006
Angiosperm	*Chrysothamnus nauseosus*	-0.80	-2.90	CE	diffuse-porous	Hacke et al. 2000a
Angiosperm	*Chrysothamnus viscidiflorus*	-1.10	-4.25	CE	diffuse-porous	Hacke et al. 2000a
Angiosperm	*Cistus albidus*	-2.03	-5.78	AD	diffuse-porous	Martinez-Vilalta et al. 2002
Angiosperm	*Cistus laurifolius*	-0.89	-3.65	AD	semi-ring-porous	Martinez-Vilalta et al. 2002
Angiosperm	*Cornus florida*	-2.19	-5.84	AD	diffuse-porous	Maherali et al. 2006
Angiosperm	*Dispyros texana*	-5.20	-6.80	AD	diffuse-porous	Johnson 2018
Angiosperm	*Eucalyptus crebra*	-3.98	-5.86	IM	diffuse-porous	Peters et al. in review
Angiosperm	*Eucalyptus saligna*	-4.82	-4.86	IM	diffuse-porous	Peters et al. in review
Angiosperm	*Fagus sylvatica*	-2.75	-2.74	IM	diffuse-porous	Losso et al. 2019
Angiosperm	*Grayia spinosa*	-2.20	-5.25	CE	diffuse-porous	Hacke et al. 2000a
Angiosperm	*Hymenoclea salsola*	-0.87	-2.64	AD	ring-porous	Mencuccini & Comstock 1997
Angiosperm	*Ilex aquifolium*	-1.75	-6.60	AD	diffuse-porous	Martinez-Vilalta et al. 2002
Angiosperm	*Kielmeyera coriacea*	-0.80	-1.91	AD	diffuse-porous	Domec et al. 2006a
Angiosperm	*Liquidambar styraciflua*	-1.45	-3.12	AD	diffuse-porous	Maherali et al. 2006
Angiosperm	*Nyssa sylvatica*	-1.70	-1.82	AD	diffuse-porous	Maherali et al. 2006
Angiosperm	*Olea europaea*	-7.10	-5.67	IM	diffuse-porous	Rodrigueuz-Dominguez et al. 2018

Angiosperm	*Oxydendrum arboreum*	-1.95	-4.54	AD	semi-ring-porous	Maherali et al. 2006
Angiosperm	*Phillyrea latifolia*	-5.30	-6.55	AD	diffuse-porous	Martinez-Vilalta et al. 2002
Angiosperm	*Populus balsamifera*	-1.00	-1.80	AD	diffuse-porous	Hacke & Sauter 1996
Angiosperm	*Prosopis velutina*	-1.95	-3.00	AD	diffuse-porous	Hultine et al. 2006
Angiosperm	*Prosopis velutina*	-1.54	-1.57	AD	diffuse-porous	Hultine et al. 2006
Angiosperm	*Prospis glandulosa*	-3.60	-4.50	AD	diffuse-porous	Johnson 2018
Angiosperm	*Qualea parviflora*	-1.00	-2.23	AD	diffuse-porous	Domec et al. 2006a
Angiosperm	*Quercus alba*	-1.16	-1.37	AD	ring-porous	Maherali et al. 2006
Angiosperm	*Quercus falcata*	-0.81	-0.92	AD	ring-porous	Maherali et al. 2006
Angiosperm	*Quercus fusiformis*	-1.30	-1.80	AD	ring-porous	Johnson 2018
Angiosperm	*Quercus fusiformis*	-0.57	-0.50	AD	ring-porous	McElrone et al. 2004
Angiosperm	*Quercus ilex*	-1.23	-2.02	DH	diffuse-porous	Martinez-Vilalta et al. 2002
Angiosperm	*Quercus ilex*	-5.00	-6.80	DH	ring-porous	Rodiguez-calcerrada 2016
Angiosperm	*Quercus nigra*	-0.86	-1.31	AD	ring-porous	Maherali et al. 2006
Angiosperm	*Quercus palustris*	-3.13	-4.56	IM	diffuse-porous	Peters et al. in review
Angiosperm	*Quercus phellos*	-1.24	-1.42	AD	ring-porous	Maherali et al. 2006
Angiosperm	*Quercus rubra*	-1.15	-1.61	AD	ring-porous	Maherali et al. 2006
Angiosperm	*Rhamnus californica*	-0.30	-2.51	CE	diffuse-porous	Pratt et al. 2007a, b
Angiosperm	*Rhamnus californica*	-1.43	-2.02	CE	diffuse-porous	Pratt et al. 2008
Angiosperm	*Rhamnus crocea*	-2.07	-5.17	CE	semi-ring-porous	Pratt et al. 2007a, b
Angiosperm	*Rhamnus crocea*	-1.44	-5.68	CE	semi-ring-porous	Pratt et al. 2008
Angiosperm	*Rhamnus ilicifolia*	-2.34	-5.92	CE	semi-ring-porous	Pratt et al. 2007a, b
Angiosperm	*Rhamnus ilicifolia*	-2.38	-5.09	CE	semi-ring-porous	Pratt et al. 2008
Angiosperm	*Sideroxylon lanuginosum*	-0.73	-2.60	AD	diffuse-porous	McElrone et al. 2004
Angiosperm	*Solanum lycopersicum*	-1.38	-1.58	IM		Skelton et al. 2017
Angiosperm	*Sorbus torminalis*	-0.86	-3.18	AD	diffuse-porous	Martinez-Vilalta et al. 2002
Angiosperm	*Tetradymia glabrata*	-2.50	-5.50	CE	diffuse-porous	Hacke et al. 2000a

Angiosperm	*Ulmus minor*	-3.00	-3.20	DH	ring-porous	Rodiguez-calcerrada 2016
Gymnosperm	*Abies balsamea*	-2.80	-3.79	CE	vesselless	Hacke & Jansen 2009
Gymnosperm	*Abies balsamea*	-1.60	-3.33	CE	vesselless	Hacke & Jansen 2009
Gymnosperm	*Abies balsamea*	-1.59	-4.49	CE	vesselless	Hacke & Jansen 2009
Gymnosperm	*Abies concolor*	-3.40	-8.40	CE	vesselless	Hacke et al. 2004
Gymnosperm	*Abies lasiocarpa*	-3.30	-3.30	CE	vesselless	Hacke et al. 2004
Gymnosperm	*Abies lasiocarpa*	-3.14	-4.70	CE	vesselless	Pittermann et al. 2006a, b
Gymnosperm	*Agathis australis*	-1.10	-2.58	CE	vesselless	Pittermann et al. 2006a, b
Gymnosperm	*Cedrus deodara*	-6.33	-6.77	IM	vesselless	Peters et al. in review
Gymnosperm	*Dacrydium cupressiformis*	-1.90	-3.08	CE	vesselless	Pittermann et al. 2006a, b
Gymnosperm	*Juniperus arizonica*	-9.50	-13.80	CE	vesselless	Willson et al. 2008
Gymnosperm	*Juniperus ashei*	-12.95	-13.41	CE	vesselless	McElrone et al. 2004
Gymnosperm	*Juniperus ashei*	-4.48	-13.41	CE	vesselless	McElrone et al. 2004
Gymnosperm	*Juniperus ashei*	-9.40	-13.10	CE	vesselless	Willson et al. 2008
Gymnosperm	*Juniperus barbadensis*	-7.20	-12.80	CE	vesselless	Willson et al. 2008
Gymnosperm	*Juniperus deppeana*	-8.80	-8.90	CE	vesselless	Willson et al. 2008
Gymnosperm	*Juniperus flaccida*	-7.80	-7.80	CE	vesselless	Willson et al. 2008
Gymnosperm	*Juniperus lucayana*	-6.30	-8.30	CE	vesselless	Willson et al. 2008
Gymnosperm	*Juniperus maritima*	-6.35	-7.58	CE	vesselless	Ogle et al 2009
Gymnosperm	*Juniperus monosperma*	-9.30	-11.60	CE	vesselless	Willson et al. 2008
Gymnosperm	*Juniperus occidentalis*	-8.50	-9.00	CE	vesselless	Willson et al. 2008
Gymnosperm	*Juniperus osteosperma*	-5.65	-6.92	AD	vesselless	Linton et al. 1998
Gymnosperm	*Juniperus osteosperma*	-7.03	-7.81	CE	vesselless	Pittermann et al. 2006a, b
Gymnosperm	*Juniperus osteosperma*	-10.40	-11.90	CE	vesselless	Willson et al. 2008
Gymnosperm	*Juniperus pinchotii*	-7.70	-14.10	CE	vesselless	Willson et al. 2008
Gymnosperm	*Juniperus scopulorum*	-5.20	-7.30	CE	vesselless	Hacke et al. 2004
Gymnosperm	*Juniperus scopulorum*	-4.96	-8.99	CE	vesselless	Ogle et al 2009

Gymnosperm	*Juniperus scopulorum*	-6.60	-7.70	CE	vesselless	Willson et al. 2008
Gymnosperm	*Juniperus virginiana*	-4.54	-7.12	CE	vesselless	Maherali et al. 2006
Gymnosperm	*Juniperus virginiana*	-4.90	-5.80	CE	vesselless	Willson et al. 2008
Gymnosperm	*Juniperus virginiana*	-4.70	-6.60	CE	vesselless	Willson et al. 2008
Gymnosperm	*Picea engelmanii*	-5.10	-4.91	CE	vesselless	Pittermann et al. 2006a, b
Gymnosperm	*Picea engelmannii*	-5.00	-8.70	CE	vesselless	Hacke et al. 2004
Gymnosperm	*Picea mariana*	-4.46	-4.59	CE	vesselless	Hacke & Jansen 2009
Gymnosperm	*Picea mariana*	-3.30	-5.33	CE	vesselless	Hacke & Jansen 2009
Gymnosperm	*Picea mariana*	-2.17	-5.98	CE	vesselless	Hacke & Jansen 2009
Gymnosperm	*Picea mariana*	-5.10	-5.98	CE	vesselless	Pittermann et al. 2006a, b
Gymnosperm	*Pinus caribaea*	-1.91	-3.27	CE	vesselless	Pittermann et al. 2006a, b
Gymnosperm	*Pinus contorta*	-2.16	-3.67	CE	vesselless	Pittermann et al. 2006a, b
Gymnosperm	*Pinus echinata*	-1.47	-3.21	AD	vesselless	Maherali et al. 2006
Gymnosperm	*Pinus edulis*	-4.20	-7.00	CE	vesselless	Hacke et al. 2004
Gymnosperm	*Pinus halepensis*	-0.88	-3.11	AD	vesselless	Oliveras et al. 2003
Gymnosperm	*Pinus monophylla*	-3.74	-5.55	CE	vesselless	Pittermann et al. 2006a, b
Gymnosperm	*Pinus pinea*	-1.01	-3.65	AD	vesselless	Oliveras et al. 2003
Gymnosperm	*Pinus ponderosa*	-1.26	-2.65	AD	vesselless	Domec et al. 2004
Gymnosperm	*Pinus ponderosa*	-1.07	-2.79	AD	vesselless	Stout & Sala 2003
Gymnosperm	*Pinus ponderosa*	-0.91	-3.43	AD	vesselless	Stout & Sala 2003
Gymnosperm	*Pinus sylvestris*	-1.65	-2.30	AE	vesselless	Mencuccini et al 1997
Gymnosperm	*Pinus taeda*	-1.74	-3.13	AD	vesselless	Maherali et al. 2006
Gymnosperm	*Pinus taeda*	-1.23	-3.27	CE	vesselless	Ewers et al. 2000
Gymnosperm	*Pinus taeda*	-2.10	-3.20	CE	vesselless	Hacke et al. 2000b
Gymnosperm	*Pinus taeda*	-0.90	-3.70	CE	vesselless	Hacke et al. 2000b
Gymnosperm	*Prumnopitys ferruginea*	-4.34	-5.58	CE	vesselless	Pittermann et al. 2006a, b
Gymnosperm	*Pseudotsuga menziesii*	-1.20	-3.30	AD	vesselless	Domec et al. 2006b, 2009

Gymnosperm	*Pseudotsuga menziesii*	-3.91	-5.21	AD	vesselless	Kavanagh et al. 1999
Gymnosperm	*Pseudotsuga menziesii*	-3.88	-5.35	AD	vesselless	Kavanagh et al. 1999
Gymnosperm	*Pseudotsuga menziesii*	-3.34	-5.15	AD	vesselless	Kavanagh et al. 1999
Gymnosperm	*Pseudotsuga menziesii*	-3.07	-5.26	AD	vesselless	Kavanagh et al. 1999
Gymnosperm	*Pseudotsuga menziesii*	-2.53	-4.77	AD	vesselless	Kavanagh et al. 1999
Gymnosperm	*Pseudotsuga menziesii*	-3.93	-4.60	AD	vesselless	Sperry & Ikeda 1997
Gymnosperm	*Pseudotsuga menziesii*	-1.75	-5.04	AD	vesselless	Sperry & Ikeda 1997
Gymnosperm	*Pseudotsuga menziesii*	-2.65	-4.49	AD	vesselless	Stout & Sala 2003
Gymnosperm	*Pseudotsuga menziesii*	-1.45	-5.25	AD	vesselless	Stout & Sala 2003
Gymnosperm	*Sequoia sempervirens*	-6.28	-6.04	CE	vesselless	Pittermann et al. 2006a, b
Gymnosperm	*Taxodium distichum*	-0.80	-2.14	CE	vesselless	Pittermann et al. 2006a, b

References

Alder, N.N., J. S. Sperry, and W. T. Pockman. 1996. Root and stem xylem embolims, stomatal conductance and leaf turgor in *Acer grandidentatum* populations along a soil moisture gradient. Oecologia 105: 293–301.

Alder, N.N., Pockman W.T., Sperry J.S. and Nuismer S. 1997. Use of centrifugal force in the study of xylem cavitation. Journal of Experimental Botany 48: 665–674.

Domec J-C., Scholz F.G., Bucci S.J., Meinzer F.C. Goldstein G., Villalobos-Vega R. 2006. Diurnal and seasonal variation in root xylem embolism in tropical savanna woody species: relationship to stomatal behavior and plant water potential. Plant Cell and Environment 29: 26-35.

Domec, J.-C., B. Lachenbruch, and F. C. Meinzer. 2006. Bordered pit structure and function determine spatial patterns of air-seeding thresholds in xylem of Douglas-fir (*Pseudotsuga menziesii*; Pinaceae) trees. American Journal of Botany 93: 1588-1600.

Domec J.-C., Warren J.M., Meinzer F.C., Lanchenbruch B. 2009. Safety factors for xylem failure by implosion and air-seeding within roots, trunks and branches of young and old conifer trees. IAWA Journal 30: 101-120.

Domec, J. C., Warren, J. M., Meinzer, F. C., Brooks, J. R., & Coulombe, R. (2004). Native root xylem embolism and stomatal closure in stands of Douglas-fir and ponderosa pine: mitigation by hydraulic redistribution. Oecologia, 141, 7-16.

Ewers B.E., Oren R. and Sperry J.S. 2000. Influence of nutrient versus water supply on hydraulic architecture and water balance in *Pinus taeda*. Plant, Cell and Environment 23: 1055–1066.

Hacke U.G. & Jansen S. 2009. Embolism resistance of three boreal conifer species varies with pit structure. New Phytologist 182: 675–686.

Hacke, U. G., J. S. Sperry, and J. Pittermann. 2000. Drought experience and cavitation resistance in six desert shrubs of the Great Basin, Utah. Basic and Applied Ecology. 1:31–41.

Hacke, U. G., J. S. Sperry, B. E. Ewers, D. S. Ellsworth, K. V. R. Schäfer, and R. Oren. 2000. Influence of soil porosity on water use in Pinus taeda. Oecologia 124: 495–505.

Hacke U., Sperry J.S., Pockman W.T., Davis S.D. and McCulloh K.A. 2001. Trends in wood density and structure are linked to prevention of xylem implosion by negative pressure. Oecologia 126: 457–461.

Hacke U.G., Sperry J.S. & Pittermann J. 2004. Analysis of circular bordered pit function II. Gymnosperm tracheids with torus-margo pit membranes. American Journal of Botany 91: 386-400.

Hultine, K. R., D. F. Koepke, W. T. Pockman, A. Fravolini, J. S. Sperry, and D. G. Williams. 2006. Influence of soil texture on hydraulic properties and water relations of a dominant warm-desert phreatophyte. Tree Physiology 26:313-323.

Johnson, D.M., Domec, J.C., Carter Berry, Z., Schwantes, A.M., McCulloh, K.A., Woodruff, D.R., Wayne Polley, H., Wortemann, R., Swenson, J.J., Scott Mackay, D. and McDowell, N.G., 2018. Co-occurring woody species have diverse hydraulic strategies and mortality rates during an extreme drought. Plant, Cell & Environment 41: 576-588.

Kavanagh K.L., Bond B.J., Aitken S.N., Gartner B.L. & Knowe S. 1999. Shoot and root vulnerability to xylem cavitation in four populations of Douglas-fir seedlings. Tree Physiology 19: 31-37.

Linton, M.J., J. S. Sperry, and D. G. Williams. 1998 Limits to water transport in *Juniperus osteosperma* and Pinus edulis: implications for drought tolerance and regulation of transpiration. Functional Ecology 12:906–911.

Losso, A., Bär, A., Dämon, B., Dullin, C., Ganthaler, A., Petruzzellis, F., Savi, T., Tromba, G., Nardini, A., Mayr, S. and Beikircher, B. 2019. Insights from in vivo micro-CT analysis: testing the hydraulic vulnerability segmentation in *Acer pseudoplatanus* and *Fagus sylvatica* seedlings. New Phytologist: 1831-1842.

Maherali H., Moura C.F., Caldeira M.C., Willson C.J. & Jackson R.B. 2006. Functional coordination between leaf gas exchange and vulnerability to xylem cavitation in temperate forest trees. Plant, Cell and Environment, 29: 571-583.

Martínez-Vilalta J, Prat E, Oliveras I and Piñol J. 2002. Xylem hydraulic properties of roots and stems of nine Mediterranean woody species. Oecologia 133: 19-29.

McElrone, A.J., W.T. Pockman, J. Martínez-Vilalta and R.B. Jackson. 2004. Variation in xylem structure and function in stems and roots of trees to 20 m depth. New Phytologist. 163:507-517.

Mencuccini, M., and J. Comstock. 1997. Vulnerability to cavitation in populations of two desert species, *Hymenoclea salsola* and *Ambrosia dumosa*, from different climatic regions. Journal of Experimental Botany 48:1323–1334.

Mencuccini M, Grace J, Fioravanti M. 1997. Biomechanical and hydraulic determinants of tree structure in Scots pine: anatomical characteristics. Tree Physiology 17: 105-113.

Ogle K., Barber J., Willson C. & Thompson B. 2009. Hierarchical statistical modeling of xylem vulnerability to cavitation. New Phytologist 182: 541–554

Oliveras I., Martínez-Vilalta J., Jimenez-Ortiz T., Lledó M.J., Escarré A. and Piñol J. 2003. Hydraulic properties of *Pinus halepensis, Pinus pinea* and *Tetraclinis articulata* in a dune ecosystem of Eastern Spain. Plant Ecology 169: 131-141.

Pittermann J., Sperry J.S., Hacke U.G., Wheeler J.K., Sikkema E.L. 2006. Inter-tracheid pitting and the hydraulic efficiency of conifer wood: the role of tracheid allometry and cavitation protection. American Journal of Botany 93: 1265–1273.

Pittermann J., Sperry J.S., Wheeler J.K., Hacke U.G., Sikkema E. 2006. Mechanical reinforcement against tracheid implosion compromises the hydraulic efficiency of conifer xylem. Plant, Cell and Environment 29: 1618-1628.

Pratt RB, Jacobsen AL, Golgotiu KA, Sperry JS, Ewers FW, Davis SD. 2007. Life history type coupled to water stress tolerance in nine Rhamnaceae species of the California chaparral. Ecological Monographs 77: 239-253.

Pratt RB, Jacobsen AL, Ewers FW, Davis SD. 2007. Relationships among xylem transport, biomechanics, and storage in stems and roots of nine Rhamnaceae species of the California chaparral. New Phytologist 174: 787-798.

Pratt RB, Jacobsen AL, Mohla R, Ewers FW, Davis SD. 2008. Linkage between water stress tolerance and life history type in seedlings of nine chaparral species (Rhamnaceae). Journal of Ecology 96: 1252-1265

Rodríguez-Calcerrada, J., Li, M., López, R., Cano, F.J., Oleksyn, J., Atkin, O.K., Pita, P., Aranda, I. and Gil, L., 2017. Drought-induced shoot dieback starts with massive root xylem embolism and variable depletion of nonstructural carbohydrates in seedlings of two tree species. New Phytologist 213: 597-610.

Skelton, R.P., Brodribb, T.J. and Choat, B., 2017. Casting light on xylem vulnerability in an herbaceous species reveals a lack of segmentation. New Phytologist: 561-569.

Sperry J.S. & Hacke U.G. 2002. Desert shrub water relations with respect to soil characteristics and plant functional type. Functional Ecology 16: 367-378.

Sperry J.S. & Hacke U.G. 2004. Analysis of circular bordered pit function I. Angiosperm vessels with homogenous pit membranes. American Journal of Botany 91: 369-385.

Sperry, J. S., and T. Ikeda. 1997. Xylem cavitation in roots and stems of Douglas-fir and white fir. Tree Physiology 17:275–280.

Sperry, J. S., and N. Z. Saliendra. 1994. Intra-and Inter-plant variation in xylem cavitation in *Betula occidentalis*. Plant, Cell and Environment 17:1233–1241.

Stout D.L. & Sala A. 2003. Xylem vulnerability to cavitation in *Pseudotsuga menziesii* and *Pinus ponderosa* from contrasting habitats. Tree Physiology 23, 43–50.

Tsuda, M., and M. T. Tyree. 1997. Whole plant hydraulic resistance and vulnerability segmentation in *Acer saccharinum*. Tree Physiology 17:351–357.

Willson CJ, PS Manos, RB Jackson. 2008. Hydraulic traits are influenced by phylogenetic history in the drought-resistant and invasive genus *Juniperus* (Cupressaceae). American Journal of Botany 95:299-314.
